# LncRNA LINC02574 Inhibits Influenza A Virus Replication by Positively Regulating the Innate Immune Response

**DOI:** 10.3390/ijms24087248

**Published:** 2023-04-14

**Authors:** Yanwei Zhang, Xiaojuan Chi, Jingyun Hu, Shulin Wang, Senhong Zhao, Yanan Mao, Benqun Peng, Jilong Chen, Song Wang

**Affiliations:** 1 Key Laboratory of Animal Pathogen Infection and Immunology of Fujian Province, Fujian Agriculture and Forestry University, Fuzhou 350002, China; 2 Key Laboratory of Fujian-Taiwan Animal Pathogen Biology, Fujian Agriculture and Forestry University, Fuzhou 350002, China

**Keywords:** influenza A virus, long noncoding RNA, interferon, viral replication, innate immunity

## Abstract

Studies have shown that long noncoding RNAs (lncRNAs) play crucial roles in regulating virus infection, host immune response, and other biological processes. Although some lncRNAs have been reported to be involved in antiviral immunity, many lncRNAs have unknown functions in interactions between the host and various viruses, especially influenza A virus (IAV). Herein, we demonstrate that the expression of lncRNA LINC02574 can be induced by IAV infection. Treatment with viral genomic RNA, poly (I:C), or interferons (IFNs) significantly stimulated LINC02574 expression, while RIG-I knockdown and IFNAR1 knockout significantly decreased LINC02574 expression after viral infection or IFN treatment. In addition, inhibition of LINC02574 expression in A549 cells enhanced IAV replication, while overexpression of LINC02574 inhibited viral production. Interestingly, knockdown of LINC02574 attenuated the expression of type I and type III IFNs and multiple ISGs, as well as the activation of STAT1 triggered by IAV infection. Moreover, LINC02574 deficiency impaired the expression of RIG-I, TLR3, and MDA5, and decreased the phosphorylation level of IRF3. In conclusion, the RIG-I-dependent interferon signaling pathway can induce LINC02574 expression. Moreover, the data reveal that LINC02574 inhibits IAV replication by positively regulating the innate immune response.

## 1. Introduction

Influenza A virus (IAV) is an RNA virus belonging to the Ormyxoviridae family [1]. IAV is airborne and primarily spreads through the air, causing respiratory disease in humans, poultry, and wild animals. Furthermore, IAV easily undergoes point mutations and genetic recombination, leading to the emergence of new strains and periodic pandemics [2]. IAV has caused many pandemics worldwide, posing a serious threat to human and animal health [3,4,5,6].

Innate immunity is the body’s first line of defense against viral invasion. IAV is first recognized by pattern recognition receptors (PRRs), including retinoic acid-inducible gene I (RIG-I), melanoma differentiation-associated gene 5 (MDA5), and toll-like receptor 3 (TLR3) [7,8,9,10]. The activation of PRRs immediately activates the downstream signaling pathway, finally inducing the transcription of interferon (IFN) genes [11]. IFNs bind to their corresponding receptors and induce the expression of antiviral proteins encoded by IFN-stimulated genes (ISGs) [12].

Long noncoding RNAs (lncRNAs) have a transcript length exceeding 200 nt and lack protein-coding ability. There are more lncRNAs in whole-genome transcription products than coding RNAs. Although lncRNAs do not encode any proteins, their expression is specific in different tissues and developmental stages. LncRNA plays a key role in the regulation network of various life activities by interacting with DNA, RNA, and protein, or regulating other coding gene expression from various aspects, such as chromatin remodeling, transcriptional regulation, and post-transcriptional processing [13,14,15,16].

LncRNA exerts its functions mainly through the following mechanisms: (1) LncRNA is a competitive endogenous RNA (ceRNA) that leads to a loss of or reduction in miRNA function and controls the regulation of miRNA on target genes [17]. (2) LncRNA activates or inhibits certain cellular activities by binding to some epigenetic-related proteins, thus modifying specific protein translation after transcription [18]. (3) LncRNA inhibits gene transcription by directly binding to chromosomal DNA, or promoting gene transcription through the recruitment of transcription factors [19]. (4) LncRNA acts as the precursor of miRNA [20]. 

LncRNAs are increasingly recognized as critical regulators of antiviral innate immune responses, including PRR-related signaling, the production of IFNs and inflammatory cytokines, JAK-STAT signaling, and the transcription of antiviral ISGs. For example, lncRNA NRAV acts as a negative regulator of the host antiviral response by affecting the histone modification of several critical ISGs and suppressing the transcription of those ISGs [21]. Besides processing miR-155, MIR155HG-derived lncRNA-155 also directly modulates host innate immunity during virus infection via regulation of TLR3 and protein-tyrosine phosphatase 1B (PTP1B)-mediated IFN response [22,23]. Other studies have also demonstrated that lnc-ISG20 acts as a competing endogenous RNA that binds to miR-326 to reduce the inhibition of ISG20 mRNA translation, thus inhibiting IAV replication [24]. 

In this study, we characterized the expression and function of a previously described lncRNA LINC02574 (also known as HEAL) [25,26] in influenza virus infection. We found that the expression of LINC02574 was significantly upregulated during IAV infection. IFNs induced the expression of LINC02574 through the RIG-I/IFN/IFNAR pathway. Importantly, LINC02574 inhibited IAV replication by enhancing IFN signaling. Our study provides a new insight into the role of LINC02574 in the regulation of host antiviral innate immune response.

## 2. Results

### 2.1. Influenza Virus Infection Upregulates LINC02574 In Vitro

**Figure 1:**
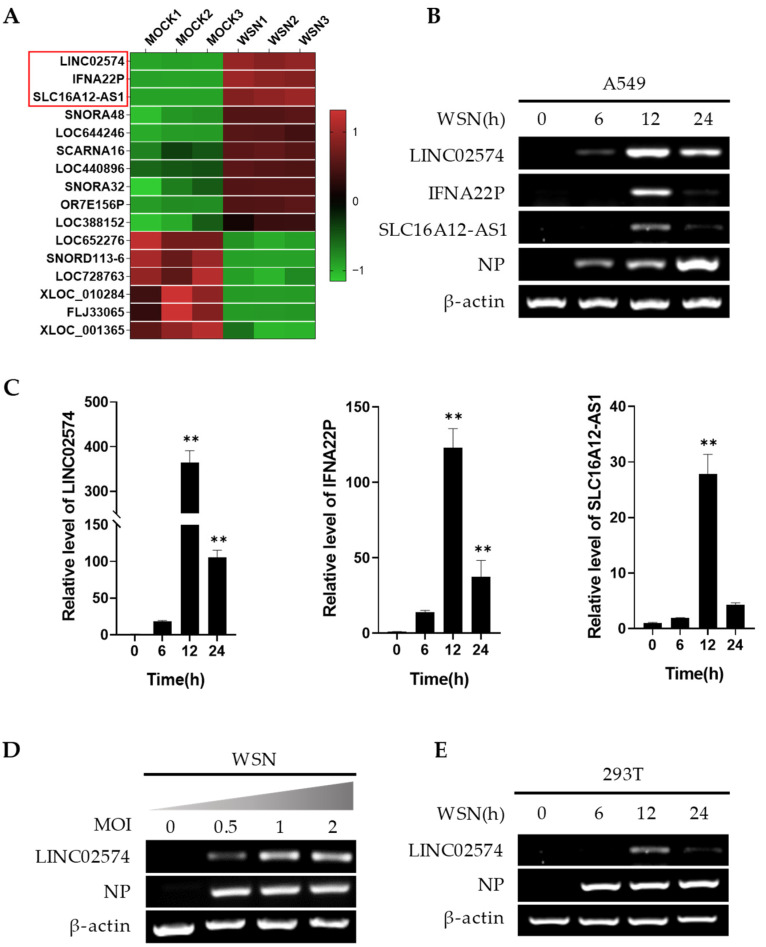
The expression of LINC02574 is significantly upregulated in response to IAV infection. (**A**) The differentially expressed lncRNAs in A549 cells infected with or without WSN influenza virus were analyzed by a cDNA microarray in our previous study (GEO accession no. GSE58741). Shown are representative lncRNAs whose expressions significantly changed after viral infection. Three lncRNAs with significant changes (fold change > 10) are indicated in a red rectangle. (**B**,**C**) Differential expressions of three selected lncRNAs in A549 cell infected with or without WSN were examined by reverse transcriptase-polymerase chain reaction (RT-PCR) and quantitative real time-polymerase chain reaction (qRT-PCR). (**D**) A549 cells were infected with different multiplicities of infection of WSN for 12 h. The expression level of LINC02574 was detected by RT-PCR. (**E**) The expression of LINC02574 in 293T cells infected with WSN was detected by RT-PCR. Data are represented as mean ± SD; *n* = 3; One-way analysis of variance (ANOVA) followed by LSD test: ** *p* < 0.01 versus time 0 h.

The LncRNA microarray performed in our previous study on human lung epithelial A549 cells infected with or without influenza A virus (GEO accession no. GSE58741) was further screened and analyzed to explore the role of host lncRNAs in influenza virus infection and replication [27]. However, only 10 upregulated and 6 downregulated lncRNAs of 1051 differentially expressed lncRNAs were screened and analyzed (Figure 1A). Three lncRNAs with significant expression changes (fold change > 10) were selected for verification via reverse transcription-PCR (RT-PCR) and quantitative RT-PCR (qRT-PCR) (Figure 1B,C). A/WSN/33 influenza virus (WSN) infection upregulated mRNA expression levels of the three lncRNAs in A549 cells, especially LINC02574 (Figure 1B,C). A549 cells were infected with WSN of different multiplicities of infection (MOI), and WSN significantly upregulated LINC02574 in a concentration-dependent manner (Figure 1D). In addition, RT-PCR results showed that influenza virus infection also induced the expression of LINC02574 in 293T cells (Figure 1E). These results demonstrate that influenza virus infection can significantly induce LINC02574 expression in human A549 and 293T cell lines.

### 2.2. Multiple Influenza Virus Strains Can Induce LINC02574 Expression

**Figure 2:**
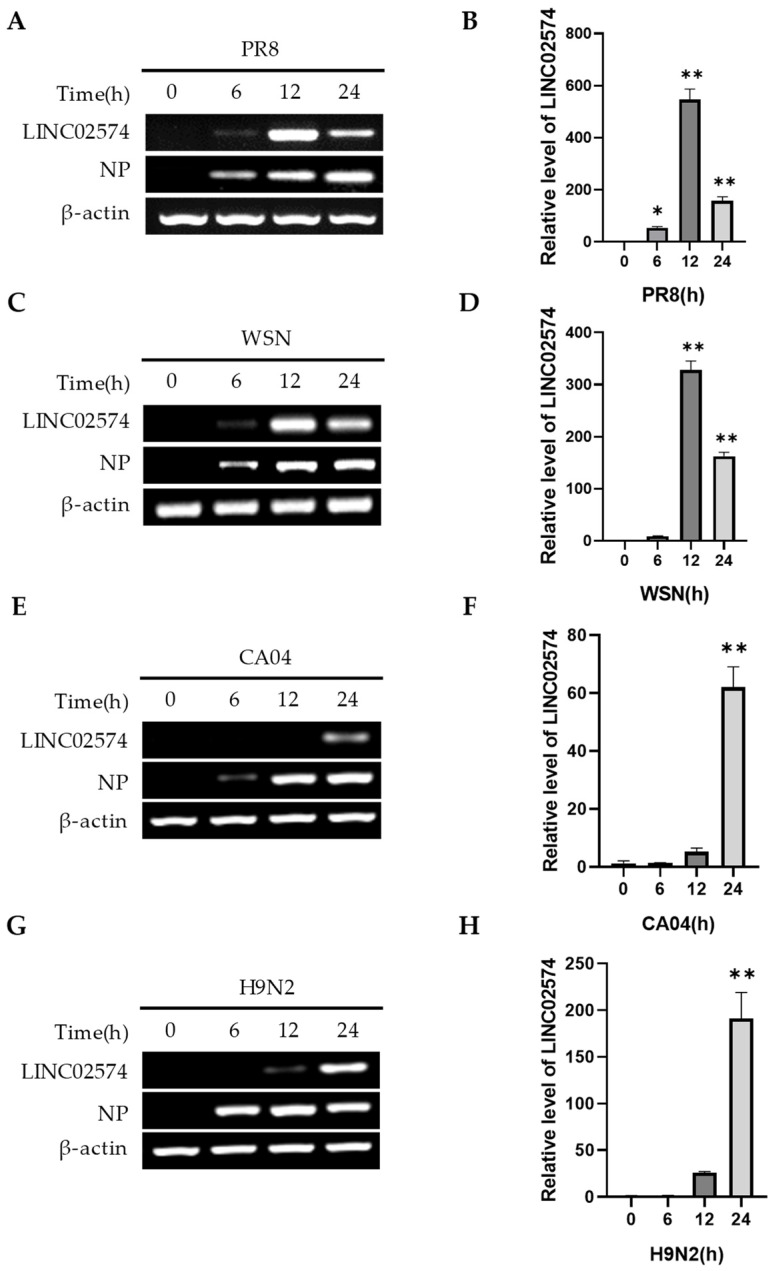
LINC02574 is induced by multiple virus infections. (**A**–**H**) The expression levels of LINC02574 in A549 cells infected with IAV PR8 (**A**,**B**), WSN (**C**,**D**), CA04 (**E**,**F**), and H9N2 (**G**,**H**) were examined by RT-PCR and qRT-PCR. Data are represented as mean ± SD; *n* = 3; One-way ANOVA followed by LSD test: * *p* < 0.05, ** *p* < 0.01 versus time 0 h.

The A549 cells were infected with different subtypes or strains of IAV for 0, 6, 12, and 24 h to determine the effect of different viral infections on LINC02574 expression. RT-PCR and qRT-PCR results showed that H1N1- (WSN, PR8, CA04) and H9N2-subtype influenza viruses induced LINC02574 expression (Figure 2A–H). However, various influenza virus strains had different effects on the dynamic expression of LINC02574. Notably, LINC02574 expression was highest after PR8 and WSN infections at 12 h (Figure 2A–D), while the expression of LINC02574 after CA04 and H9N2 influenza virus infections was highest at 24 h (Figure 2E–H). These results indicate that LINC02574 is an IAV-induced lncRNA that can be upregulated by multiple influenza virus strains in A549 cells.

### 2.3. IFNs Regulate LINC02574 Expression

**Figure 3:**
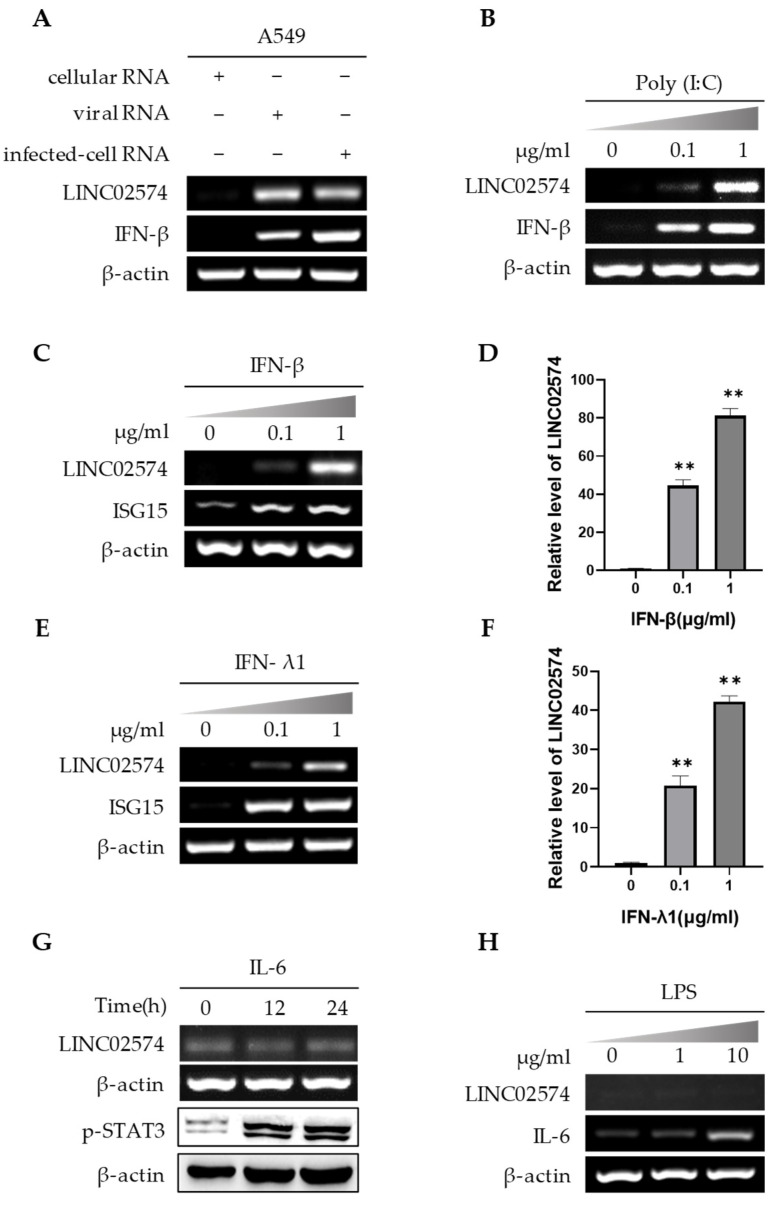
LINC02574 is an IFN-stimulated gene. (**A**) Cellular RNA (total RNA from uninfected control A549 cells), viral RNA, and infected-cell RNA (total RNA from A549 cell infected with PR8 virus) were transfected into A549 cells for 24 h. RT-PCR was performed to examine the expression level of LINC02574. (**B**) A549 cells were transfected with poly(I:C) at the indicated concentrations for 18 h. The expression levels of LINC02574 were examined by RT-PCR. (**C**–**F**) A549 cells were treated with IFN-β (**C**,**D**) or IFN-λ1 (**E**,**F**) at indicated concentrations for 12 h, and the expression of LINC02574 was examined by RT-PCR (**C**,**E**) and qRT-PCR (**D**,**F**). (**G**) A549 cells were treated with IL-6 (0.1 μg/mL) for the indicated times. The expression of LINC02574 was detected by RT-PCR and the phosphorylation level of STAT3 was examined by Western blotting. (**H**) A549 cells were incubated with LPS at the indicated concentrations for 12 h. The expression of LINC02574 was determined by RT-PCR. Data are represented as mean ± SD; *n* = 3; One-way analysis of variance (ANOVA) followed by LSD test: ** *p* < 0.01 versus 0 μg/mL.

The A549 cells were first transfected with the genomic RNA of PR8 influenza virus (viral RNA) and the total RNA of cells infected with PR8 influenza virus (infected-cell RNA) to further explore the virus-induced LINC02574 expression. RNA of cells without virus infection was used as the control (cellular RNA). RT-PCR results showed that viral RNA and infected-cell RNA significantly upregulated LINC02574 (Figure 3A). Poly(I:C) is a synthetic analogue of viral double-stranded RNA. Poly(I:C) transfection in A549 cells also induced the expression of LINC02574 in a dose-dependent manner (Figure 3B). Both influenza viral RNA and poly(I:C) are potent inducers of IFNs, which indicates that virus-induced IFNs can upregulate LINC02574 expression. Furthermore, type I interferon IFN-β and type III interferon IFN-λ1 significantly upregulated LINC02574 in A549 cells in a dose-dependent manner (Figure 3C–F). In addition, influenza virus RNA can induce the expression of inflammatory factors. However, both IL-6 and lipopolysaccharide (LPS) (inducer of inflammatory factors) treatment did not affect LINC02574 expression (Figure 3G,H). These results indicate that IFNs may play a key role in the upregulation of LINC02574 after viral infection.

### 2.4. Influenza Virus Upregulates LINC02574 through RIG-I/IFN/IFNAR Signaling Pathway

**Figure 4:**
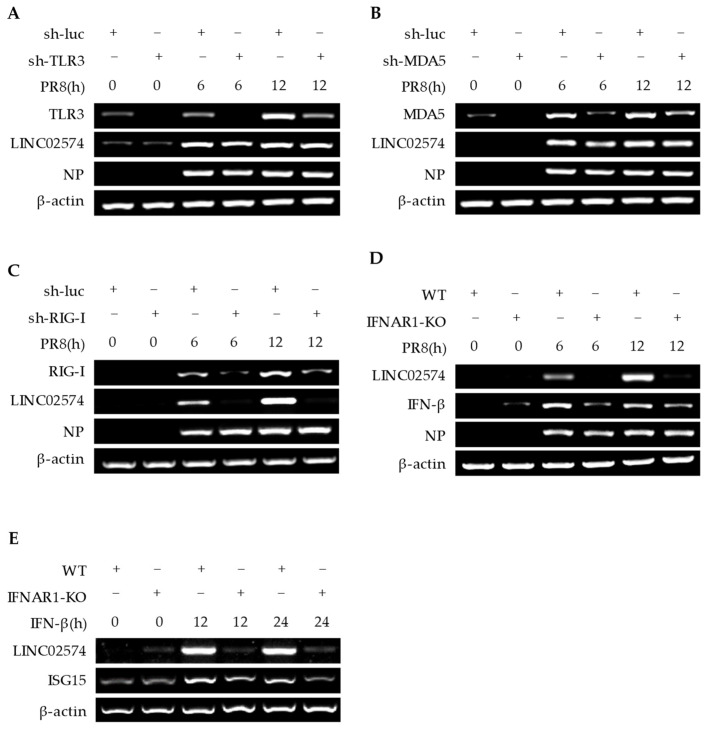
IAV-induced LINC02574 expression is regulated by RIG-I/IFN/IFNAR signaling. (**A**) The expression levels of LINC02574 in TLR3 knockdown and sh-luc control cells infected with or without PR8 virus were determined by RT-PCR. (**B**) The expression levels of LINC02574 in MDA5 knockdown and sh-luc control cells infected with or without PR8 virus were determined by RT-PCR. (**C**) The expression levels of LINC02574 in RIG-I knockdown and sh-luc control cells infected with or without PR8 virus were determined by RT-PCR. (**D**) LINC02574 levels in IFNAR1 knockout or control cells infected with or without PR8 virus were detected by RT-PCR. (**E**) LINC02574 levels in IFNAR1 knockout or control cells treated with or without IFN-β were detected by RT-PCR. *n* = 3.

Some conserved components produced during the replication of influenza virus, such as pathogen-associated molecular patterns (PAMPs), are recognized by the host’s PRRs, triggering a series of signaling cascades, resulting in the production of IFNs and proinflammatory cytokines [28]. RIG-I, MDA5, and TLR3 are the main PRRs that recognize influenza virus PAMPs. Herein, A549 cell lines that can stably knock down RIG-I, MDA5, and TLR3 were developed using small hairpin RNA (shRNA) interference to further explore the IFN-induced signaling pathway involved in LINC02574 expression. The effects of RIG-I, MDA5, and TLR3 knockdown on the LINC02574 expression in A549 cells were first detected. The results showed that knockdown of MDA5 and TLR3 did not affect LINC02574 expression after influenza virus infection, while RIG-I knockdown significantly downregulated LINC02574 induced by influenza virus (Figure 4A–C).

IFNs exert their antiviral effect by binding to receptors on the surface of cell membranes and activating the expression of a series of antiviral ISGs. In this study, a type I IFN receptor knockout (IFNAR1 KO) cell model of A549 cells was stimulated by influenza virus infection or IFN-β for further analysis. Influenza virus or IFN-induced LINC02574 expression were significantly reduced in IFNAR1 KO cells compared with control cells (Figure 4D,E). These results suggest that influenza virus infection induces LINC02574 expression through the regulation of the RIG-I/IFN/IFNAR signaling pathway.

### 2.5. LINC02574 Inhibits the Replication of Influenza Virus

**Figure 5:**
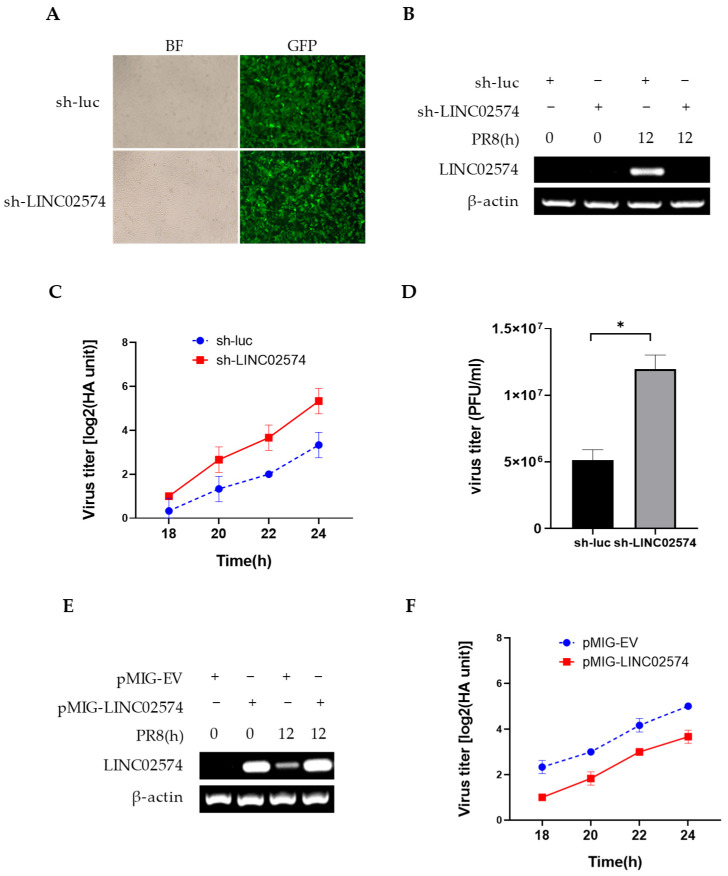
LINC02574 inhibits the replication of IAV. (**A**) Shown are A549 cell lines stably expressing shRNAs targeting LINC02574 (sh-LINC02574) or luciferase control (sh-luc). (**B**–**D**) A549 cells stably expressing specific shRNAs targeting LINC02574 or luciferase control were infected with PR8 virus for 24 h. Total RNA was then extracted for RT-PCR to detect the expression of LINC02574 (**B**). The cell culture supernatants were harvested at the indicated times for hemagglutinin assay (**C**) and at 20 h for plaque-forming assay (**D**) to measure virus titers. (**E**,**F**) A549 cells carrying either LINC02574-expressing plasmid (pMIG-LINC02574) or empty vector (pMIG-EV) were infected with or without PR8 virus for 24 h. After infection, total RNA was extracted for RT-PCR to detect LINC02574 expression (**E**). The cell culture supernatants were harvested at the indicated times for hemagglutinin assay (**F**). Data are represented as mean ± SD; *n* = 3; * *p* < 0.05, Student’s *t*-test.

In this study, we also evaluated whether LINC02574 is involved in the regulation of influenza virus replication. A549 cells stably expressing shRNA targeting LINC02574 or luciferase control were generated. LINC02574 or luciferase shRNA were expressed by lentivirus using vector pSIH-H1-GFP, which encodes a GFP reporter in addition to the shRNA. When A549 cells were infected with luciferase shRNA-containing lentivirus (sh-luc), versus sh-LINC02574, it was found that infection efficiency was similar in both cell lines (Figure 5A). The expression level of LINC02574 was significantly decreased in sh-LINC02574 cells compared with that in sh-luc control cells (Figure 5B). The cells were then infected with PR8 influenza virus, and the virus titer in cell supernatant was detected via hemagglutinin assay and plaque-forming assay. We observed a small but significantly increased virus titer in LINC02574 knockdown cells compared to that in control cells (Figure 5C,D), indicating that LINC02574 deficiency increases the replication of influenza virus. Further, LINC02574 overexpression in A549 cells led to a small but significantly decreased virus titer (Figure 5E,F). 

### 2.6. Knockdown of LINC02574 Attenuates the Innate Immune Response to Influenza Virus Infection

**Figure 6:**
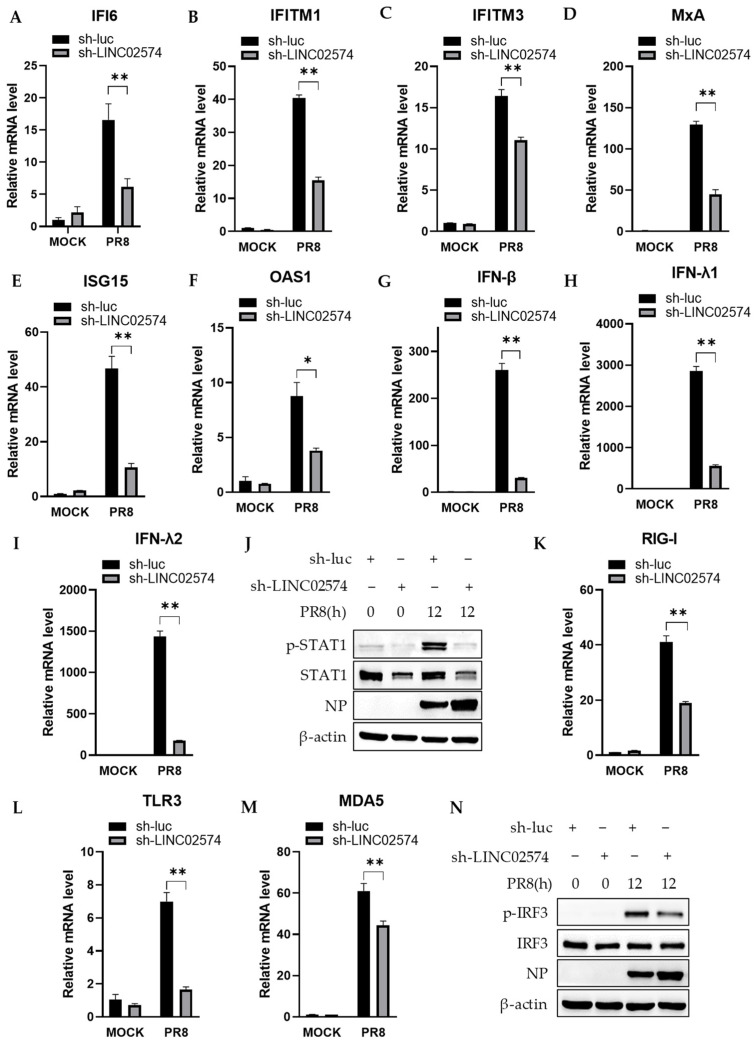
LINC02574 knockdown attenuates the innate immune response to influenza virus. (**A**–**N**) A549 cells stably expressing specific shRNAs targeting LINC02574 or luciferase control were infected with PR8 virus for 24 h. After infection, the mRNA expressions of IFI6 (**A**), IFITM1 (**B**), IFITM3 (**C**), MxA (**D**), ISG15 (**E**), OAS1 (**F**), IFN-β (**G**), IFN-λ1 (**H**), IFN-λ2 (**I**), RIG-I (**K**), TLR3 (**L**), MDA5 (**M**) were detected by qRT-PCR. The phosphorylation levels of STAT1 (**J**) and IRF3 (**N**) were examined by Western blotting. Data are represented as mean ± SD; *n* = 3; * *p* < 0.05, ** *p* < 0.01, Student’s *t*-test.

LncRNA mainly regulates the expression of its neighboring genes. Herein, we examined the expression of IFI6, an ISG located upstream and adjacent to LINC02574. Consistent with the expression of LINC02574, IFI6 mRNA expression was significantly upregulated in A549 cells with PR8 influenza virus infection. However, a significant decrease in IFI6 mRNA expression was observed in LINC02574 knockdown cell lines compared with control cell lines (Figure 6A). To determine if LINC02574 is uniquely involved in IFI6 mRNA expression, we detected the mRNA levels of several other ISGs that play a key role in antiviral immunity. The results showed that LINC02574 knockdown also significantly downregulated PR8-induced IFITM1, IFITM3, MxA, ISG15, and OAS1 (Figure 6B–F). These results indicate that LINC02574 regulates the mRNA expression of multiple ISGs that may contribute to the antiviral effects of LINC02574.

LINC02574 depletion led to a decrease in the mRNA level of multiple ISGs nonspecifically, suggesting that it may impact the IFN-related signaling pathway. To test the hypothesis, we first explored the effect of LINC02574 on the production of IFNs. As predicted, the silencing of LINC02574 in A549 cells significantly suppressed the mRNA expression of IFN-β, IFN-λ1, and IFN-λ2 (Figure 6G–I). The transcription factor STAT1 plays an essential role in antiviral innate immunity in the response to type I and III IFNs. Next, we examined the activation of STAT1 in LINC02574 knockdown cells and control cells. LINC02574 knockdown resulted in significantly decreased activation and expression of STAT1 (Figure 6J). Since LINC02574 depletion impaired IFN production and STAT1 activation, this prompted us to further investigate the effect of LINC02574 on the expression of RIG-I, TLR3, and MDA5, the three important PRRs that recognize the invading influenza virus and mediate the production of IFNs. As shown in Figure 6K–M, knockdown of LINC02574 significantly impaired the expression of RIG-I, TLR3, and MDA5 on the mRNA level. In addition, the phosphorylation level of IRF3 was strongly attenuated in LINC02574 knockdown cells compared with the control cells (Figure 6N). These results suggest that LINC02574 can inhibit the replication of influenza virus by positively regulating the innate immune responses.

## 3. Discussion

Several studies have demonstrated that lncRNA can regulate gene expression at multiple levels, such as epigenetic, transcriptional, post-transcriptional, and translational levels. LncRNA can regulate gene expression at the epigenetic level through histone modification and chromatin remodeling [14]. LncRNA can interact with transcription factors or coactivation factors at the transcription level to regulate gene expression [15]. LncRNA can regulate gene expression at the post-transcriptional level by regulating mRNA stability and acting as ceRNAs [16,29]. In addition, lncRNA can regulate expression at the translation level by directly binding to mRNA and other pathways [30,31].

As potent regulatory molecules, lncRNAs play key roles in various biological processes, including virus–host interaction. Many studies have shown that lncRNAs participate in the host antiviral immune response by regulating ISG expression. For example, lncRNA IFITM4P acts as a ceRNA to regulate some members of the IFITM family and inhibits the replication of influenza A virus [17]. A primate-specific lncRNA CHROMR can restrict viral infection of macrophages by sequestering the IFN regulatory factor (IRF)-2-dependent transcriptional corepressor IRF2BP2, thereby promoting the transcription of the ISG network [32]. Besides facilitating ISGs expression, lncRNA can also negatively regulate ISGs. LncRNA LUCAT1 inhibits the transcription of ISGs by interacting with STAT1 in the nucleus and restraining the innate immune response in host cells [33].

In this study, knockdown of LINC02574 in A549 cells attenuated the expression of multiple ISGs and type I and type III IFNs, as well as the activation of STAT1 triggered by IAV infection, indicating that LINC02574 positively regulates the innate immune response to viral infection. Consistently, our data reveal that LINC02574 functions as an antiviral host factor against IAV infection. Overexpression of LINC02574 inhibited the replication of IAV, while knockdown of LINC02574 promoted IAV replication. However, a previous study described LINC02574 as HIV-1-enhanced lncRNA (HEAL), which is upregulated by HIV-1 infection. HEAL interacts with the RNA-binding protein FUS to enhance transcriptional coactivator p300 recruitment to the HIV promoter and positively regulate HIV transcription, thus facilitating HIV replication in T cells and macrophages [25]. Therefore, LINC02574 may play different roles by targeting different pathways in different viral infections. It can not only inhibit viral replication by boosting the host innate immune response, but can also be hijacked by the virus to enhance viral replication.

RIG-I, TLR3, and MDA5 are critical PRRs for sensing RNA viruses. After infection, the PRRs recognize viral RNAs, and then trigger the IFN signaling pathway to enable effective defense against invading pathogens. LncRNAs can regulate the host antiviral immune response through interactions with the PRRs. For example, Lnczc3h7a binds to both TRIM25 and activated RIG-I, stabilizes the RIG-I–TRIM25 interaction, and thus promotes downstream signaling transduction [34]. lnc-Lsm3b competes with viral RNA for binding to RIG-I, then inhibits RIG-I activation and prevents overproduction of type I IFNs [35]. LncITPRIP-1 binds to the C terminus of MDA5 and promotes the oligomerization and activation of MDA5, thereby enhancing the innate immune response to viral infection [36]. We show here that knockdown of LINC02574 attenuates the expression of RIG-I, TLR3, and MDA5 and impairs the activation of IRF3 and the induction of IFNs triggered by IAV infection, indicating that LINC02574 may act as a switch to activate antiviral immune responses by regulating PRR signaling.

In addition, different subtypes or strains of IAV can induce the expression of LINC02574. The IFN signal regulates the expression of LINC02574, which in turn augments the production of IFNs and multiple ISGs. These results indicate that LINC02574 may be involved in a broad-spectrum antiviral innate immune response. However, only the function of LINC02574 in the replication of IAV was examined. Therefore, future studies should verify the function of LINC02574 using more viruses and determine whether LINC02574 has a broad-spectrum antiviral function in the host.

## 4. Materials and Methods

### 4.1. Cell Lines and Cell Culture

The A549, 293T, and MDCK cell lines were sourced from American Type Culture Collection (Manassas, VA, USA). The cells were cultured in Dulbecco’s modified Eagle’s medium (DMEM) (Gibco, Grand Island, NY, USA) supplemented with 10% heat-inactivated fetal bovine serum (Gibco, USA), penicillin (100 U/mL), and streptomycin (100 μg/mL) at 37 °C under a humidified 5% CO_2_ atmosphere as previously described [37].

Short hairpin RNA (shRNA)-based knockdown cell lines were generated through the infection of A549 or 293T cells with lentiviruses expressing specific shRNAs in pSIH-H1-GFP vector as previously described [38]. The following sequences were used in the shRNAs targeting specific genes: LINC02574 shRNA, 5′-CATACAGACGGACGGATAAAT-3′ and luciferase control shRNA, 5′-CTTACGCTGAGTACTTCGA-3′.

The A549 cells stably expressing LINC02574 were generated through infection with retroviruses encoding the lncRNA in pMIG vector. IFNAR1 knockout cell line was generated using a regularly clustered interspaced short palindromic repeats-associated protein 9 (CRISPR-Cas9) system as previously described [39].

### 4.2. Viruses and Viral Infection

Influenza A virus strains, including A/WSN/1933 (H1N1), A/Puerto Rico/8/1934 (H1N1), A/California/04/2009 (H1N1), and A/Chicken/Fujian/MQ01/2015 (H9N2), were propagated in specific-pathogen-free (SPF) embryonated chicken eggs, as previously described [40]. The cells were infected with viruses at the indicated multiplicity of infection (MOI). The cells were washed with phosphate-buffered saline (PBS) after adsorption for 1 h at 37 °C and cultured in DMEM containing 2 μg/mL of trypsin.

### 4.3. Plaque Forming Assay and Hemagglutinin Assay

MDCK cells were infected with serially diluted cell culture supernatants at 37 °C for 1 h. The cells were then washed with PBS and overlaid with α-minimal essential medium containing 3% low-melting-point agarose and 2 μg/mL TPCK (L-1-tosylamido-2-phenylethyl chloromethyl ketone)-treated trypsin. The cells were fixed with 4% formaldehyde after incubation at 37 °C for 72 h. The plaques were then stained with 1% crystal violet hydrate solution and counted. For hemagglutinin assay, the supernatants were diluted with PBS and mixed with an equal volume of 1% chicken erythrocytes. The viral titers were counted from the highest dilution factors that produced a positive reading.

### 4.4. RT-PCR and Quantitative Real-Time PCR (qRT-PCR)

Trizol reagent (Invitrogen, Carlsbad, CA, USA) was used to extract total RNA from cells. cDNA was synthesized using 5 mg of total RNA, oligo-dT primers or random primers, and reverse transcriptase (RT; Promega, Madison, WI, USA). PCR and quantitative PCR were then conducted using rTaq DNA polymerase and SYBR PremixEx TaqII (TaKaRa, Tokyo, Japan), respectively. β-actin was used as a reference gene for internal standardization. qPCR data were expressed as normalized ratios autocalculated using DDCT method via LightCycler system (Roche, Basel, Switzerland).

### 4.5. Western Blotting

Cell lysates were prepared, and Western blotting was performed as previously described [40]. Briefly, samples were separated by sodium dodecyl sulfate-polyacrylamide gel electrophoresis (SDS-PAGE) and transferred onto a nitrocellulose membrane in a wet tank transfer system. Blots were blocked in TBS buffer (10 mM Tris–HCl, pH 7.4, 150 mM NaCl) containing 5% skim milk for 1 h at room temperature, and then probed with antibodies as indicated. The protein bands were visualized by chemiluminescence using the FluorChem E System (ProteinSimple, San Jose, CA, USA). The following antibodies were used in this study: anti-STAT1 and anti-phospho-STAT1 (Tyr701), anti-phospho-STAT3 (Tyr705) (Cell Signaling Technology, Boston, MA, USA); anti-IRF3 and anti-phospho-IRF3 (Ser386) (Abcam, Cambridge, UK); anti-IAV NP (produced in our lab); anti-β-actin (TransGen Biotech, Beijing, China).

### 4.6. Statistical Analysis

Student’s *t*-test was used for comparison between two groups. One-way analysis of variance (ANOVA) followed by LSD test was used for multiple comparisons. Data are represented as mean ± SD (standard deviation). *p* < 0.05 was considered a statistically significant difference.

## Data Availability

All raw data are available upon reasonable request.
